# Characterizing the Time-Perspective of Nations with Search Engine Query Data

**DOI:** 10.1371/journal.pone.0095209

**Published:** 2014-04-15

**Authors:** Takao Noguchi, Neil Stewart, Christopher Y. Olivola, Helen Susannah Moat, Tobias Preis

**Affiliations:** 1 Department of Psychology, University of Warwick, Coventry, United Kingdom; 2 Tepper School of Business, Carnegie Mellon University, Pittsburgh, Pennsylvania, United States of America; 3 Warwick Business School, University of Warwick, Coventry, United Kingdom; University of Maribor, Slovenia

## Abstract

Vast quantities of data on human behavior are being created by our everyday internet usage. Building upon a recent study by Preis, Moat, Stanley, and Bishop (2012), we used search engine query data to construct measures of the time-perspective of nations, and tested these measures against per-capita gross domestic product (GDP). The results indicate that nations with higher per-capita GDP are more focused on the future and less on the past, and that when these nations do focus on the past, it is more likely to be the distant past. These results demonstrate the viability of using nation-level data to build psychological constructs.

## Introduction

As individuals increasingly rely on the Internet to plan for the future and retrieve information on past events, online activity provides new insights into both human interests and behavior. Aggregated data now offered by Internet services such as Google, Yahoo, Wikipedia and Flickr open up new possibilities to investigate both temporal and spatial differences in the information sought and distributed by users around the globe (e.g., [1]–[9]). Previously, online information flow has been associated with real world human behavior, in particular in the economic domain (e.g., [10]–[17]). Here, we use aggregated data on searches retrieved from Google Trend, with insights from the psychology of individuals, to construct nation-level measures of a psychological characteristic. In particular, we take time-perspective and examine its association with per-capita gross domestic product (GDP).

Time-perspective is one of the most extensively researched characteristics at the level of individuals, and previous research reports its close association with the economic activity of individuals. For instance, individuals who consider future events are more likely to have clearly defined career goals and to sacrifice present enjoyment to achieve these goals [18]. Individuals who consider future events more frequently than current events are also less likely to show various impulsive behaviors [19] and aggressive behavior [20]. We therefore expect a nation's tendency to focus on the future to have a positive association with per-capita GDP. Indeed, an association between time-perspective and per-capita GDP is suggested by Preis, Moat, Stanley, and Bishop [16].

**Figure 1:**
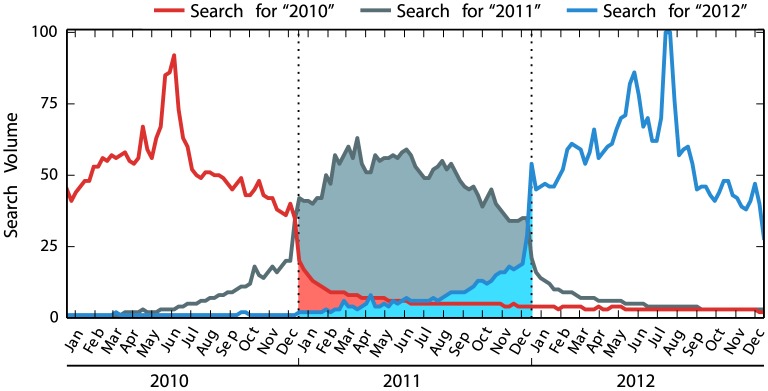
Search volume, provided by Google Trends, for the United Kingdom in 2010–2012.

To measure time-perspective, we followed Preis et al. [16], who examined the relative volume of, for example, searches for the terms “2010” and “2012” made during the year 2011. Figure 1 plots the frequency of searches for “2010”, “2011” and “2012” over time, normalized (by Google Trends) to have a maximum of 100 for the period between 2010 and 2012. The blue-shaded area shows weekly search volume for “2012” made during 2011, which indicates the extent to which a nation is focused on the future. Similarly, the red-shaded area indicates the extent to which a nation is focused on the past.

Preis et al. [16] calculated the ratio of the blue-shaded area (i.e., searches for the upcoming year) to the red-shaded area (i.e., searches for the previous year). This ratio, termed the “future orientation index”, correlates with per-capita GDP, suggesting that a nation with higher per-capita GDP is more likely focused on the future relative to the past.

Here, we address a key question: Is higher per-capita GDP associated with a greater focus on the future, a lesser focus on the past, or both? Future focus measures the extent to which a nation was focused on the future, relative to the present, and is quantified as the ratio of the blue-shaded area to the gray-shaded area in Figure 1, where the gray-shaded area shows the weekly search volume for the present year. Past focus is measured as the ratio of the red-shaded area to the gray-shaded area.

Unlike future focus, the extent to which individuals focus on the past does not have a clear connection to their behavior [21]. However Preis et al. 's [16] findings suggest that past focus may have a negative relationship with economic activity. We therefore use our new measures to test this relationship.

In addition to examining focus on past and future events, as described above, we also examine the degree to which an individual searches for near rather than more distant events. Psychological studies on time-perspective show that if an individual considers events in the distant future more often than those in the near future, this individual exhibits impulsive behavior less frequently [22], [23]. In addition, various impulsive behaviors are associated with the extent to which an individual discounts consequences of his or her decision in the distant future more than consequences in the near future. For example, greater discounting is associated with smoking, illicit drug use, higher body mass index, and recent infidelity. Discounting is also associated with demographic variables, with younger, poorer, less educated individuals discounting the distant future to a greater extent (for review, see [24]).

We therefore investigate the effects of a second form of time-perspective: time-horizon. An individual's time-horizon is defined as how far into the future or past he or she tends to think. Although the effects of past time-horizon have not been investigated as extensively as the effect of future time-horizons, past time-horizon is expected to have the same effects as future time-horizon [25].

A nation's time-horizon is measured as the steepness of the curves in Figure 1. For instance, the steep increase in searches for “2012” toward the end of the year 2011 indicates that the near future is much more frequently searched than the distant future: i.e., the future time-horizon is short. Similarly, the steep decrease in searches for “2010” from the beginning of the year 2011 indicates a short past time-horizon.

In summary, we construct four measures of time-perspective: future focus, past focus, future time-horizon, and past time-horizon. Future focus, future time-horizon and past time-horizon are predicted to have a positive relationship with per-capita GDP, whereas past focus is predicted to have a negative relationship.

## Method

From Google Trends (http://trends.google.com), we retrieved weekly search volumes for six different years expressed in Arabic numerals: 2007, 2008, 2009, 2010, 2011 and 2012. The use of Arabic numerals allows cross-linguistic comparison. We then constructed the four measures as described above. We first summed weekly search volumes to derive annual search volumes. The future focus is calculated as the ratio of the annual volumes for the immediately following year (i.e., the red-shaded area in Figure 1) to the annual volumes for the present year (i.e., the gray-shaded area). The past focus is calculated as the ratio of the annual volumes for the immediately preceding year (i.e., the blue-shaded area) to the annual volumes for the present year.

**Figure 2:**
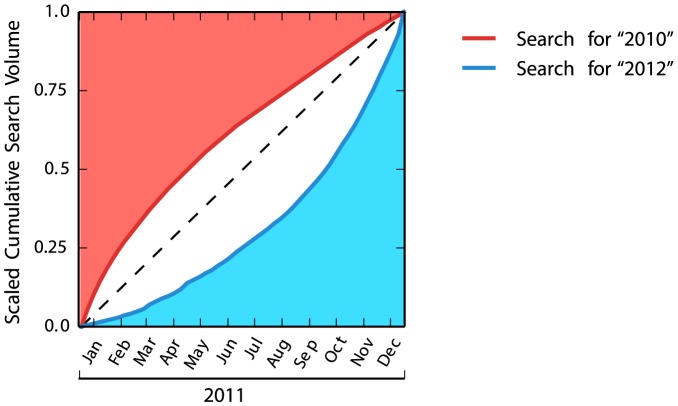
Scaled cumulative search volume for the United Kingdom. The red shaded area corresponds to the past time-horizon, and the blue shaded area corresponds to the future time-horizon.

The future horizon is a measure of the rate at which searches for the immediately following year pick up within a given year. Similarly, the past horizon is a measure of the rate at which searches for the immediately preceding year drop off within a given year. These measures are derived from the cumulative distribution of search volume. First, we divided weekly search volume by annual search volume, so that cumulative search volume sums to 1. This scaling allows us to compare how search volume changes whilst controlling for the differences in overall search volume between nations. We then quantified the difference between scaled cumulative search volume and constant search volume. Constant search volume is a hypothetical search volume, where the future/past is searched throughout the year at a constant rate, and where overall search volume sums to 1. An example is illustrated in Figure 2. The blue line represents a running total of the search volume for the term “2012” throughout the year 2011, for the United Kingdom. The line is initially flatter than constant search (the dashed line) and then becomes steeper. This convexity indicates that there are fewer “2012” searches early in 2011. The more convex the scaled cumulative search volume is, the more rapidly search volume increases at the end of the year. When the scaled cumulative search volume is more convex, the blue-shaded area is smaller. Thus, a smaller blue-shaded area indicates a shorter future time-horizon.

Likewise, the red-shaded area measures the past time-horizon. The concavity of the red line in Figure 2 indicates that there are more searches for the term “2010” early in the year 2011. The more concave the scaled cumulative search volume is, the more rapidly the search volume decreases from the beginning of the year. When the scaled cumulative search volume is more concave, the red-shaded area is smaller, indicating a shorter past time-horizon.

These four scores vary slightly across years, depending on the day that the first full week of the year started. For instance, the first full week of the year starts on January 3rd in 2010 but on January 2nd in 2011. As data from Google Trends are only available at a weekly scale for most of the search terms, the search volume on January 2nd is included in the scores for 2011 but excluded in the scores for 2010, which results in a larger past focus score for 2011 than for 2010. This is because the search volume for the past year tends to be larger early in the year (e.g., January 2nd) than at later dates in the year. To reduce this artefactual variance between years, we rescaled the four scores independently for each of the four years, so that a measured score ranges from 0 to 1 in a given year.

The four scores were computed for the 43 nations with more than five million Internet users, as reported in the CIA World Factbook as of 1 July 2010, and whose per-capita GDPs from 2008 to 2011 were available from the Word Bank in February 2013 (see Table S1 for the full list of nations). Per-capita GDP is positively skewed and therefore log-transformed.

Following the suggestion of Simmons, Nelson, and Simonsohn [26], we declare that the data we report here are the entirety of the data we collected and that the results we report below do not depend on arbitrary analytic decisions (e.g., whether to rescale the four scores to range from 0 to 1). In addition, all datasets we use are freely available online, so that our analyses can be easily verified and extended by other interested researchers.

## Results

To test the reliability of our four time-perspective measures, we first examine their temporal stability. Auto-correlation coefficients are .85 (95% CI [.80, .89]) for the future focus, .77 (95% CI [.68, .83]) for the past focus, .74 (95% CI [.65, .81]) for the future time-horizon, and .62 (95% CI [.50, .72]) for the past time-horizon at the annual resolution and at one-year lag. These coefficients indicate that our measures are relatively stable and reliable across time.

**Figure 3:**
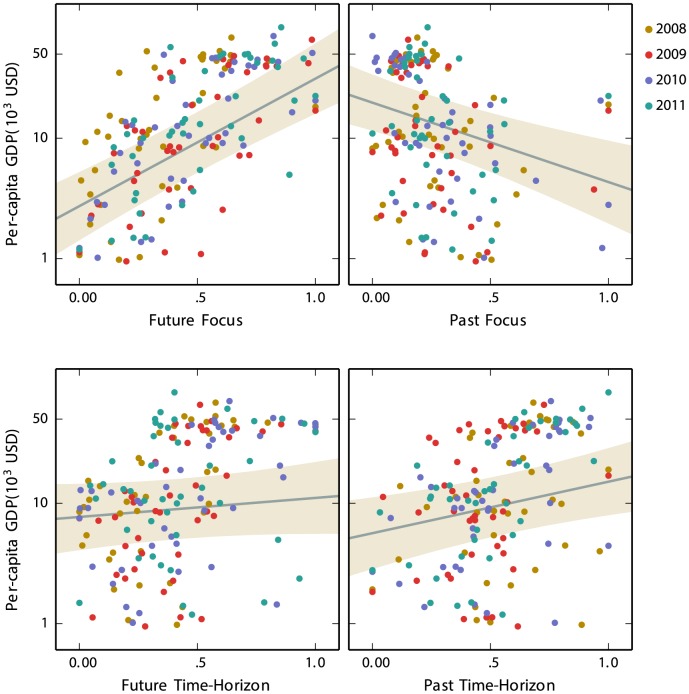
Per-capita GDP as a function of future and past focuses and future and past time-horizons. The solid lines represent predictions from the mixed-effect model, and the shaded areas represent 95% confidence regions of the predictions, based on the intercept and the given fixed effect. In plotting the model prediction, the other fixed effects (e.g., the effect of past focus in the top left panel) are marginalized by mean-averaging.

Figure 3 displays per-capita GDP as a function of our time-perspective measures. For example, the top left panel plots per-capita GDP against future focus. Each point represents a nation in a particular year. The vertical axis is on the log scale and indicates the per-capita GDP for a nation, while the horizontal axis represents the future focus for the nation in the same year. The other three panels plot per-capita GDP against past focus, future time-horizon and past time-horizon. Figure 3 shows that a nation with higher per-capita GDP tends to have a higher score for the future focus and the future and past time-horizons, but a lower score for the past focus.

To confirm statistically the relationship between these measures and per-capita GDP, we simultaneously enter these four scores as fixed factors into a mixed-effect linear model to predict logged per-capita GDP in USD. The random factors are a by-year intercept and slopes (see Text S1 for details). Model fit indicates that three of the measures are significant predictors: the future focus (χ^2^(1) = 19.65, *p*<.001), the past focus (χ^2^(1) = 9.19, *p* = .002), and the past time-horizon (χ^2^(1) = 7.77, *p* = .005). The future time-horizon is not a significant predictor: χ^2^(1) = 1.35, *p* = .246.

When the three significant measures are included in the model, the model predictions strongly correlate with per-capita GDP, *r* = .73 (95% CI [.65, .79]), explaining as much as 53% of the variance in per-capita GDP. For comparison, when the mixed-effect model only contains what Preis et al. 's [16] index and a by-year intercept and slope, the correlation between the model predictions and per-capita GDP shows the coefficient of *r* = .70 (95% CI [.61, .77]).

To quantitatively assess the advantage of our measures over Preis et al.'s [16] index, we conducted a five-fold cross-validation: we first randomly partitioned the nations into five groups, then fit the above models using the data from the four partitions at one time and tested the fitted model on the remaining partition. This fit-then-test process was repeated five times to test each of five partitions, and throughout the five partitions, the deviance of our model with the three significant measures (mean: 76.21) was consistently smaller than the deviance of the model with only Preis et al.'s index (mean: 88.45). Identical results were obtained with four-fold and six-fold cross-validations, demonstrating that our measures have a stronger association with per-capita GDP than Preis et al.'s index.

## Discussion

The enormous volume and availability of data on individuals' online behaviors and the increasing integration of online and real-world activity provides an alternative to the use of standard scales on relatively small samples of individuals for studying individuals' time-perspective. The present study is (to the best of our knowledge) the first to use the psychology of individuals to inspire the creation of nation-level measures of time-perspective. In particular, we have constructed four measures of time-perspective from search engine query data and examined their relationship with a widely-used measure of economic activity: per-capita GDP. As predicted, nations with high per-capita GDP are more focused on the future, less focused on the past, and have longer past time-horizons. Of course, the direction of causality cannot be established from this correlational analysis, but the strong association of our time-perspective measures with per-capita GDP does demonstrate that these measures are capturing something systematic about the economic activity of a nation.

Our finding that a greater future focus is associated with higher per-capita GDP is consistent with studies of individuals. Just as the individuals focused on the future are better able to pursue career goals [18], the nations focused on the future may be better able to strive for economic success and thus have higher per-capita GDP. The association between a greater past focus and lower per-capita GDP, however, does not follow as clearly from studies of individuals. Psychological research merely suggests that the valence of attitudes towards the past matters in associating past focus with economic activity: Individuals with a negative attitude towards the past (e.g., regret) are less likely to work towards future rewards and more likely to engage in gambling, while those with a positive attitude towards the past (e.g., nostalgia) are less likely to engage in risky behavior [18]. Thus, it is possible that the past focus, measured in the present study, is associated primarily with a negative attitude and not with a positive attitude. Also, given the positive relationship between the past time-horizon and economic activity, a tendency to consider the distant past may be linked to positive attitude, which relates to better economic activity. Thus it may be that attitudes towards the past change from negative to positive over time, which might have produced an overall null relationship between the past focus and individual's behavior in previous research (e.g., [21]).

Our measures of time-perspective are similar to Hofstede's [27] measure of long-term orientation. This long-term orientation is one of the factors that differentiates nations especially in East Asia and is characterized by the extent to which a nation values Confucian dynamism (e.g., perseverance and thrift). Unlike our measures of time-perspective, however, Hofstede's measure does not distinguish between future and past orientation, nor is grounded in psychological studies of individuals. Nonetheless, the positive association between Hofstede's measure and economic growth [28] further supports the relationship between the time-perspective and economic activity of nations.

Lastly, the past focus and time-horizon relate to a previous study which analyzed a corpus of digitized texts. To examine how quickly the past is forgotten at the societal level, Michel, Shen, Aiden, Veres, et al. [29] examined mentions of previous years in an archive of books. Their results show that the immediate past is more frequently mentioned during the 20th century than during the 19th century, but that the distant past is less frequently mentioned during the 20th century. These findings imply that on the global scale, the past focus measured in the present study may be gradually increasing while the past time-horizon is getting shorter.

In summary, we have shown the link between the psychological characteristic of a nation and its economic activity. In particular, we have broken down Preis et al.'s [16] future orientation index and showed that greater future focus and lesser past focus are both independently associated with higher per-capita GDP and add the finding that a longer past time-horizon is also associated with higher per-capita GDP. The strong associations between the psychological measures and per-capita GDP demonstrate the viability of using nation-level data, together with psychological concepts, to understand the economic activity of nations.

## Supporting Information

Table S1 **List of nations.**
(XLSX)

Text S1 **The mixed-effect linear model.**
(DOCX)
