# Supplementary material for: Characterizing the Time-Perspective of Nations with Search Engine Query Data
Source: PLoS One. 2014 Apr 15;9(4):e95209. doi: 10.1371/journal.pone.0095209 (PMC3988161; doi:10.1371/journal.pone.0095209)
Supplement: Text S1 — The mixed-effect linear model. (DOCX) [file pone.0095209.s002.docx]

*Text S2.* The mixed-effect linear model.

The statistical model we used is expressed as below:

log(per-capita GDP on year t) = *β_FF_^(t)^* × *FF* + *β_PF_^(t)^* × *PF* + *β_FH_^(t)^* × *FH* + *β_PH_^(t)^* × *PH* + *β_0_^(t)^* + ε,

where *FF*, *PF*, *FH*, and *PH* represents future focus, past focus, future time-horizon, and past time-horizon, respectively. The superscript *(t)* indexes the year. By having a by-year intercept and slopes as the random factors, we allowed the intercept and the slopes to vary between years. Thus for example, the slope for future focus is defined as follows:

*β_FF_^(t)^ ~ Normal(β_FF_, σ_FF_^2^).*

The predictions in Figure 3 are computed as below:

log(per-capita GDP) = *β_FF_* × *FF* + *β_PF_* × *PF* + *β_FH_* × *FH* + *β_PH_* × *PH* + *β_0._*
